# AVAILABILITY AND STORAGE OF HAZARDOUS PRODUCTS IN HOUSEHOLDS IN THE METROPOLITAN REGION OF MANAUS: A POPULATION-BASED SURVEY, 2015

**DOI:** 10.1590/1984-0462/2021/39/2020130

**Published:** 2021-02-05

**Authors:** Gustavo Magno Baldin Tiguman, Marina Borges Dias de Almeida, Marcus Tolentino Silva, Tais Freire Galvao

**Affiliations:** a Universidade Estadual de Campinas, Campinas, SP, Brazil.; b Universidade de Sorocaba, Sorocaba, SP, Brazil.

**Keywords:** Poisoning, Hazardous substances, Sanitizing products, Sanitizing product storage, Household products, Health surveys, Envenenamento, Substâncias perigosas, Saneantes, Armazenamento de saneantes, Produtos domésticos, Inquéritos epidemiológicos

## Abstract

**Objective:**

The availability of hazardous products in households increases the risks of poisoning. The present study aimed to assess the frequency and associated factors of the availability and storage of hazardous products in residences in the metropolitan region of Manaus.

**Methods:**

Population-based and cross-sectional study conducted in 2015 with adults selected with three-stage probabilistic sampling. Participants were interviewed face-to-face. Prevalence ratio (PR) of the presence of hazardous products (presence of *chumbinho* [illegal anti-cholinesterase rodenticide], artisanal cleaning products, and unsafe storage of these products and medications) and 95% confidence intervals (95%CI) were calculated with Poisson regression with robust variance, weighted by the complex sampling method adopted.

**Results:**

A total of 4,001 participants was included, of which 53.0% (95%CI 51.5-54.6) reported presence of hazardous products in their households, 36.3% (95%CI 34.8-37.8) had unsafe storage, 16.2% (95%CI 15.1-17.4) had artisanal cleaning products, and 8.2% (95%CI 7.4-9.1) had *chumbinho*. Households with children ≤5 years old had safer storage (PR=0.78; 95%CI 0.71-0.86) and more artisanal products (PR=1.30; 95%CI 1.11-1.51). Presence of artisanal products was higher in lower educational levels (PR=2.20; 95%CI 1.36-3.57) and lower economic classifications (PR=1.63; 95%CI 1.25-2.13).

**Conclusions:**

Over half of the households in the metropolitan region of Manaus kept hazardous products; one-third stored them unsafely. Artisanal cleaning products and *chumbinho* were frequently present. Households with children had safer storage of products, and socioeconomic factors affected the availability of such hazardous products.

## RESUMO

**Objetivo:**

A disponibilidade de produtos perigosos em domicílios aumenta os riscos de intoxicações. Este estudo objetivou avaliar a frequência e os fatores associados à disponibilidade e armazenamento de produtos perigosos em residências da Região Metropolitana de Manaus.

**Métodos:**

Estudo transversal de base populacional realizado em 2015 com adultos selecionados por amostragem probabilística em três estágios. Os participantes foram entrevistados pessoalmente. A razão de prevalência (RP) da presença de produtos perigosos (presença de chumbinho [rodenticida anticolinesterase ilegal], produtos de limpeza artesanais e armazenamento inseguro desses produtos e de medicamentos) e intervalos de confiança de 95% (IC95%) foram calculados por regressão de Poisson com variância robusta, ponderada pela amostragem complexa adotada.

**Resultados:**

4.001 participantes foram incluídos, dos quais 53,0% (IC95% 51,5-54,6) reportaram a presença de produtos perigosos em seus domicílios, 36,3% (IC95% 34,8-37,8) apresentaram armazenamento inseguro, 16,2% (IC95% 15,1-17,4) possuíam produtos de limpeza artesanais e 8,2% (IC95% 7,4-9,1) possuíam chumbinho. Os domicílios com crianças menores de 5 anos apresentaram armazenamento mais seguro (RP=0,78; IC95% 0,71-0,86) e mais produtos artesanais (RP=1,30; IC95% 1,11-1,51). Presença de produtos artesanais foi maior em menores níveis de escolaridade (RP=2,20; IC95% 1,36-3,57) e menores classificações econômicas (RP=1,63; IC95% 1,25-2,13).

**Conclusões:**

Mais da metade dos domicílios da Região Metropolitana de Manaus possuía produtos perigosos; um terço os armazenava sem segurança. Produtos de limpeza artesanais e chumbinho estavam frequentemente presentes. Os domicílios com crianças apresentaram armazenamento mais seguro de produtos e fatores socioeconômicos afetaram a disponibilidade de tais produtos perigosos.

## INTRODUCTION

Child poisoning mostly occurs at home with products easily accessible to children.^1^ Unintentional injuries, especially those related to poisoning, are an important cause of morbidity and mortality in children and adolescents worldwide.^2^ ^,^ ^3^ ^,^ ^4^ Roughly one million unintentional exposures in children ≤5 years old were reported in the United States in 2018.^5^ Children in this age group are highly vulnerable to unintentional injuries as a result of their natural curiosity to explore their surroundings, their small stature, and their lack of perception of danger.^6^

Exposure to household cleaning products were the second most common poisoning exposures in children aged 5 or less, accounting for 11% of the reported cases in the US in 2018.^5^ These cleaning substances accounted for 10% of all poisoning calls in Australia in 2015; one-third of these cases were related to children ≤5 years old.^7^ Two thirds of child poisonings managed in a Romanian hospital between 2014 and 2016 were nonmedication related, and household chemicals were the most frequent agents.^8^ In Malaysia, from 2006 to 2015, over 90% of the poisonings occurred at home through ingestion, and approximately half of the unintentional cases occurred in children.^9^ In 2013, 73% of the patients treated for poisonings in a Brazilian Poison Control Center were children aged ≤5; cleaning products were the second most frequent cause of poisonings, which mostly occurred at home.^10^

Childhood poisonings show higher lethality in low- and middle-income countries, because they are associated with poverty and weaker market regulations.^11^ Illegal products, with hazardous substances in their formulations, also play a role in poisoning exposures among children, leading to systemic symptoms and high number of hospital admissions.^12^

Brazil lacks epidemiological data about the availability and storage of such substances, especially in underdeveloped regions. We aimed to assess the frequency of the availability and unsafe storage of hazardous products and associated factors in households in the Brazilian Amazon.

## METHOD

This was a cross-sectional and population-based study, conducted between May and August 2015, which interviewed adults living in the metropolitan region of Manaus. The present analysis is part of a survey designed to assess the offer and usage of healthcare services in the region.^13^

We selected adults ≥18 years old with probabilistic sampling in three stages, by clusters, and stratified by sex and age quotas. Sample size was calculated in 4,000 interviewees, considering the estimated adult population in this region and a conservative prevalence of healthcare services utilization.^13^

Trained interviewers with previous experience in quantitative surveys collected data at the participants’ residences using questionnaires on electronic devices after obtaining written informed consent from each participant.

The dependent variable selected for this study was the availability of hazardous products in the households with 95% confidence intervals (95%CI), which was assessed with the self-reported presence of *chumbinho* (an illegal anti-cholinesterase rodenticide) or artisanal household cleaning products (products not commercially registered by the health authority), or the unsafe storage of household cleaning products, pesticides, and medications. The presence of each isolated outcome was also investigated (presence of *chumbinho*, artisanal household cleaning products, unsafe storage).

Data were obtained from the following question: “For each product listed below (household cleaning products: bleach, fabric softener, disinfectant, detergents, soap, caustic soda; *chumbinho* for rats; insecticides; and medications), are they stored in your household?” (yes or no). In case of positive answer, the following questions were asked regarding each product: “Is it artisanal?” (yes or no) - to determine whether the product was considered illegal - and “Is it stored in a high place or locked up?” (yes or no) - to determine unsafe storage.

Independent variables included the municipality (capital [Manaus] or interior towns); number of dwellers in each household (1-2, 3-5, ≥6); presence of children ≤5 years old living in the household (yes or no); interviewee’s educational level (higher education degree or above, complete high school, complete elementary school, less than elementary school); economic classification (A/B, C, D/E, according to the Brazilian Economic Classification Criteria,14 in which A refers to the wealthiest, and E, to the poorest); and visit from the Family Health Program (a Brazilian primary care program of the Unified Health System [Sistema Único de Saúde, SUS] based on care for the whole family that includes household visits) over the last 12 months (yes or no).

The list of products was asked randomly in each interview to avoid bias of desensitization of the interviewees when there was similarity between the response options, especially in the latter questions.^15^

The data were analyzed descriptively and adjusted for covariates using Poisson regression with robust variance. All variables were included in the adjusted multivariate analysis as covariates. We calculated prevalence ratio (PR) and 95% confidence intervals (95%CI) for the presence of hazardous products for each independent variable. Statistical significance was considered for p<0.05. We calculated deviance statistic and the Pearson’s Goodness-of-Fit test to assess the appropriateness of Poisson regression models.^16^ All analyses were performed in Stata 14.2 (StataCorp, College Station, TX) and considered the complex sample design (svy command) to properly weight the sample.

The Research Ethics Committee of Universidade Federal do Amazonas approved the project, under Report No. 974.428 dated March 3^rd^, 2015 (Certificate of Presentation for Ethical Appreciation [*Certificado de Apresentação para Apreciação Ética*] at Brazil Platform: 42203615.4.0000.5020). All participants signed an informed consent form.

## RESULTS

We included 4,001 participants, of which 87.0% lived in Manaus City (Table 1). Most households had 3-5 dwellers in the same residence, no children ≤5 years old, and had not received a visit from the Family Health Program over the last 12 months before interview. Most interviewees had an educational level up to high school and belonged to the middle class (economic classification C).

**Table 1:** Characteristics of participants and presence of hazardous products, unsafe storage, artisanal products, and *chumbinho* in households (Metropolitan Region of Manaus, 2015; n=4,001).

	Population frequency		Presence in the household							
			Hazardous		Unsafe storage		Artisanal		*Chumbinho*	
	n	%	n	%	n	%	n	%	n	%
Municipality										
Manaus	3,479	87.0	1,869	55.1	1,280	36.8	577	16.6	295	8.5
Interior towns	522	13.1	195	39.6	135	25.9	57	10.9	34	6.5
Number of dwellers										
1-2	854	21.3	384	45.9	290	34.0	101	11.8	45	5.3
3-5	2,274	56.8	1,255	56.7	868	38.2	379	16.7	215	9.5
≥6	873	21.8	425	50.9	257	29.4	154	17.6	69	7.9
Children ≤5 years old										
No	2,418	60.4	1,267	53.9	937	38.8	339	14.0	200	8.3
Yes	1,098	39.6	797	52.0	478	30.2	295	18.6	129	8.2
Educational level										
Higher education or above	158	4.0	82	52.6	60	37.7	17	10.8	13	8.2
High school	1,903	47.6	966	52.1	705	37.1	238	12.5	176	9.3
Elementary school	1,185	29.6	576	50.6	413	34.9	157	13.2	80	6.8
Less than elementary school	755	18.9	440	59.8	237	31.4	223	29.5	60	8.0
Economic classification										
A/B	629	15.7	342	56.1	264	42.0	67	10.7	76	12.1
C	2,285	57.1	1,161	52.6	827	36.2	324	14.2	186	8.1
D/E	1,087	27.2	561	52.6	324	29.8	243	22.4	67	6.2
Visit by Family Health Program agents*										
Yes	1,277	31.9	696	57.5	219	17.2	481	37.7	105	8.2
No	2,724	68.1	1,368	51.2	415	15.2	934	34.3	224	8.2

*In the previous 12 months.

Availability of hazardous products was self-reported in 53.0% (95%CI 51.5-54.6) of households, whereas unsafe storage was present in 36.3% (95%CI 34.8-37.8), artisanal cleaning products in 16.2% (95%CI 15.1-17.4), and *chumbinho* in 8.2% (95%CI 7.4-9.1).

In the multivariate analysis (Tables 2 and 3), living in interior towns was negatively associated with the availability of hazardous products (PR=0.68; 95%CI 0.61-0.76), unsafe storage (PR=0.68; 95%CI 0.58-0.80), and artisanal products (PR=0.56; 95%CI 0.43-0.72) when compared to living in the capital, Manaus. Households with 3-5 dwellers had more hazardous products (PR=1.24; 95%CI 1.14-1.35), unsafe storage (PR=1.16; 95%CI 1.04-1.30), artisanal products (PR=1.37; 95%CI 1.11-1.69), and *chumbinho* (PR=1.76; 95%CI 1.27-2.45). Households with children ≤5 years old presented significantly more artisanal products (PR=1.30; 95%CI 1.11-1.51) but safer storage (PR=0.78; 95%CI 0.71-0.86). Having a lower educational level (less than elementary school) was positively associated with the presence of hazardous (PR=1.22; 95%CI 1.03-1.44) and artisanal products (PR=2.20; 95%CI 1.36-3.57), whereas having a lower economic classification (D/E) was negatively associated with unsafe storage (PR=0.75; 95%CI 0.65-0.86) and the presence of *chumbinho* (PR=0.54; 95%CI 0.38-0.77), and positively associated with the presence of artisanal products (PR=1.63; 95%CI 1.25-2.13). Households not visited by the Family Health Program in the previous year had lower availability of hazardous products (PR=0.86; 95%CI 0.81-0.92), unsafe storage (PR=0.89; 95%CI 0.81-0.97), and artisanal products (PR=0.81; 95%CI 0.70-0.94). The deviance and Pearson Goodness-of-Fit tests for all regressions suggested that the adjusted models were appropriate (p>0.05).

**Table 2:** Unadjusted and adjusted prevalence ratios and 95% confidence interval of the presence of hazardous products and unsafe storage in the household (n=4,001).

	Hazardous products				Unsafe storage			
	PR (95%CI)	p-value	Adjusted PR (95%CI)	p-value	PR (95%CI)	p-value	Adjusted PR (95%CI)	p-value
Municipality								
Manaus	1.00	<0.001	1.00	<0.001	1.00	<0.001	1.00	<0.001
Interior towns	0.71 (0.63-0.79)		0.68 (0.61-0.76)		0.70 (0.60-0.82)		0.68 (0.58-0.80)	
Number of dwellers								
1-2	1.00	<0.001	1.00	<0.001	1.00	<0.001	1.00	<0.001
3-5	1.24 (1.14-1.35)		1.24 (1.14-1.35)		1.13 (1.01-1.26)		1.16 (1.04-1.30)	
≥6	1.11 (1.01-1.23)		1.10 (0.99-1.20)		0.87 (0.76-1.00)		0.96 (0.82-1.11)	
Children ≤5 years old								
No	1.00	0.315	1.00	0.059	1.00	<0.001	1.00	<0.001
Yes	0.97 (0.91-1.03)		0.94 (0.88-1.00)		0.78 (0.71-0.86)		0.78 (0.71-0.86)	
Educational level								
Higher education or above	1.00	<0.001	1.00	<0.001	1.00	0.044	1.00	0.831
High school	0.99 (0.85-1.16)		1.01 (0.86-1.18)		0.97 (0.79-1.20)		1.03 (0.84-1.27)	
Elementary school	0.96 (0.82-1.12)		1.00 (0.85-1.19)		0.91 (0.74-1.13)		1.05 (0.84-1.31)	
Less than elementary school	1.13 (0.96-1.33)		1.22 (1.03-1.44)		0.82 (0.66-1.03)		0.99 (0.78-1.26)	
Economic classification								
A/B	1.00	0.219	1.00	0.233	1.00	<0.001	1.00	<0.001
C	0.93 (0.86-1.01)		0.94 (0.87-1.02)		0.86 (0.77-0.96)		0.87 (0.78-0.97)	
D/E	0.93 (0.85-1.02)		0.92 (0.83-1.02)		0.71 (0.62-0.81)		0.75 (0.65-0.86)	
Visit by Family Health Program agents*								
Yes	1.00	<0.001	1.00	<0.001	1.00	0.035	1.00	<0.001
No	0.89 (0.84-0.95)		0.86 (0.81-0.92)		0.91 (0.83-0.99)		0.89 (0.81-0.97)	

PR: prevalence ratios; 95%CI: 95% confidence interval; *in the previous 12 months.

**Table 3:** Unadjusted and adjusted prevalence ratios and 95% confidence interval of the presence of artisanal products and *chumbinho* in the household (n=4,001).

	Artisanal products				*Chumbinho*			
	PR (95%CI)	p-value	Adjusted PR (95%CI)	p-value	PR (95%CI)	p-value	Adjusted PR (95%CI)	p-value
Municipality								
Manaus	1.00	<0.001	1.00	<0.001	1.00	0.133	1.00	0.221
Interior towns	0.62 (0.48-0.80)		0.56 (0.43-0.72)		0.77 (0.54-1.08)		0.81 (0.57-1.14)	
Number of dwellers								
1-2	1.00	0.001	1.00	0.010	1.00	0.001	1.00	0.002
3-5	1.41 (1.15-1.73)		1.37 (1.11-1.69)		1.79 (1.31-2.45)		1.76 (1.27-2.45)	
≥6	1.50 (1.19-1.89)		1.21 (0.95-1.56)		1.50 (1.04-2.16)		1.52 (1.02-2.27)	
Children ≤ 5 years old								
No	1.00	<0.001	1.00	0.001	1.00	0.880	1.00	0.386
Yes	1.38 (1.16-1.54)		1.30 (1.11-1.51)		0.98 (0.80-1.22)		0.90 (0.72-1.14)	
Educational level								
Higher education or above	1.00	<0.001	1.00	<0.001	1.00	0.094	1.00	0.250
High school	1.18 (0.74-1.88)		1.03 (0.65-1.66)		1.12 (0.65-1.92)		1.30 (0.75-2.24)	
Elementary school	1.24 (0.77-2.00)		0.99 (0.61-1,61)		0.81 (0.46-1.42)		1.05 (0.59-1.87)	
Less than elementary school	2.78 (1.75-4.43)		2.20 (1.36-3.57)		0.96 (0.54-1.71)		1.38 (0.75-2.53)	
Economic classification								
A/B	1.00	<0.001	1.00	<0.001	1.00	<0.001	1.00	0.002
C	1.33 (1.04-1.70)		1.27 (0.99-1.63)		0.67 (0.52-0.87)		0.69 (0.54-0.90)	
D/E	2.10 (1.63-2.70)		1.63 (1.25-2.13)		0.51 (0.37-0.70)		0.54 (0.38-0.77)	
Visit by Family Health Program agents*								
Yes	1.00	0.132	1.00	0.005	1.00	0.929	1.00	0.860
No	0.89 (0.77-1.04)		0.81 (0.70-0.94)		0.99 (0.79-1.24)		1.02 (0.81-1.28)	

PR: prevalence ratios; 95%CI: 95% confidence interval; *in the previous 12 months.

## DISCUSSION

The availability of hazardous and artisanal products, and unsafe storage were associated with living in the capital and having three or more dwellers in the same household. The presence of children ≤5 years old was associated with safer product storage and higher availability of artisanal cleaning products. Lower educational levels increased the availability of hazardous and artisanal products, whereas lower economic classification was associated with higher presence of artisanal products, lower availability of *chumbinho*, and safer storage of products. Visits by the Family Health Program did not reduce the availability and unsafe storage of hazardous products.

Households located in interior towns had fewer hazardous and artisanal products, and reported safer storage of cleaning products and medications. A retrospective study conducted in a Polish hospital analyzed the medical records of 848 children admitted to the institution due to poisoning exposures, from 2008 to 2012, and observed that poisoning was more frequent in urban children than in those living in rural areas,^17^ which may be explained by a lower access to these products among countryside residents.

Higher numbers of dwellers living in the same household increased the availability of hazardous products and unsafe storage. This may be a result of a higher use and access to potentially harmful products. Childhood unintentional injuries, such as burns, are more likely to occur in crowded households, as suggested by a case-control study conducted in Rio de Janeiro, Brazil, between 1991 and 1992.^18^ Another epidemiological study carried out in Greece with 1,245 children, aged up to 5 years old, indicated that child poisoning is correlated with overcrowding at home, which could lead to uncontrolled settings and, consequently, to difficulties in monitoring the child’s behavior.^19^

Households with children ≤5 years old had safer storage of cleaning products and medications, indicating a better care by guardians. Most reported cases of poisonings from a study that included five hospitals in Australia occurred in an unsupervised time span of five minutes or less, and the most common means of access were during the usage of agents, accounting for three quarters of the total cases.^20^ From 2010 to 2013, parents of over 500 poisoned children, aged 0-4 years old, in England, were less likely to store medications out of reach or safely locked, and to put medications and household products away immediately after use.^1^

Younger children are the most vulnerable group for inherent reasons, because they are prone to insert objects and liquids in their mouths, have less evolved perceptions of taste, and lack awareness about the dangers involved, which can be minimized by the usage of less appealing packages and label content for children.^21^ ^,^ ^22^ Children also have a lower body weight. Therefore, the amount of substance required to reach concentrations that can cause damage is significantly lower.

Effective measures to prevent child poisoning exposure inside their households include poison prevention education to parents, provision of emergency contact numbers of poison control centers, and safe storage of hazardous products in cupboard/drawer locks.^23^ ^,^ ^24^ When safe storage fails, other tactics may help prevent poisoning, such as improved labelling and/or packaging, and the use of safe bottles, which consist of a lid that is hard to open, requiring more strength than children usually have.^20^ ^,^ ^25^ ^,^ ^26^

Participants with lower educational levels were associated with a higher availability of hazardous and artisanal products. Lower economic classification was positively associated with the presence of artisanal products, but negatively associated with availability of *chumbinho* and unsafe storage. A Danish analysis of 173,504 children treated in emergency departments, from 1998 to 2003, observed a higher risk of unintentional home injuries in children whose mothers had lower education attainment and in those from the lowest income stratum.^27^ Plausible explanations for these differences are the awareness of dangerous practices and the development of safety habits among those with higher educational levels, and the possibility of living in safer households with reduced exposure risks among high-income families, who can afford safer equipment.^27^ Artisanal cleaning products were also more frequent in these households, which can result in higher toxicity in comparison to regulated products.^12^ A cross-sectional study that collected data from a Brazilian Poison Control Center, between 2013 and 2014, found that toxic clinical manifestations were significantly more frequent after illegal products exposure, often leading to hospitalizations and surgeries.^12^

Visits by the Family Health Program did not reduce the availability and unsafe storage of hazardous products; instead, lower presence of hazardous products, unsafe storage, and artisanal products were observed in households not visited in the previous year. In most Brazilian settings, communitarian health agents who visit households have several assignments, and are frequently underpaid, overworked, and unrecognized.^28^ This may have an impact on the level of information and assistance provided by these professionals. A qualitative study conducted in 2016 with 50 healthcare professionals from the Family Health Program showed that educational practices related to childhood poisoning promoted by these agents were mainly focused on first aids concepts, with little discussion on actual poisoning accident prevention strategies.^29^ If adopted by the Family Health Program agents, directed poisoning prevention education to parents - including increasing knowledge of poisons and poisoning prevention behaviors such as safe storage of medications and cleaning products - could possibly reduce childhood poisoning rates.^24^ Prevention with education improves poisoning prevention practices and encourages the need of legislative measures, such as those related to childproof bottles.^24^ The potential of the Family Health Program, however, may be compromised in the near future, because changes in the Brazilian Primary Healthcare Policy allowed a reduction in the size and components of Family Health Program teams.^30^

Interviews were held with one individual from each household, without objective confirmation of responses, which limits the validity of results. As the options of products available at home were repetitive, desensitization of respondents may have occurred. Such caveats may impact the assessment and represent information bias in the study. Considering the survey investigated many outcomes in only one interview, the study did not include poison prevention measures or follow-up with participants. We did not assess the incidence of poisoning of investigated products and, consequently, we cannot provide implications of the availability of hazardous products. Sample size calculations were based on estimates of health services usage, and the assessment of the outcomes of the present analysis was not planned.

In conclusion, over half households from the metropolitan region of Manaus kept hazardous products, and these products were stored unsafely in one-third of the domiciles. Artisanal cleaning products and *chumbinho* were frequently available in households. Unfavorable socioeconomic factors increased the availability of hazardous products.
